# TikTok content as a source of health education regarding epicondylitis: a content analysis

**DOI:** 10.1186/s10195-024-00757-3

**Published:** 2024-03-23

**Authors:** Riccardo D’Ambrosi, Enrico Bellato, Gianluca Bullitta, Antonio Benedetto Cecere, Katia Corona, Angelo De Crescenzo, Valentina Fogliata, Gian Mario Micheloni, Maristella Francesca Saccomanno, Fabrizio Vitullo, Andrea Celli, SICSeG (Italian Society of Shoulder, Elbow Surgery) Research Committee

**Affiliations:** 1 IRCCS Ospedale Galeazzi—Sant’Ambrogio, Milan, Italy; 2 Department of Biomedical Science for Health, University of Milan, Milan, Italy; 3 Department of Surgical Science, University of Turin, Turin, Italy; 4 San Luigi Gonzaga Hospital, Orbassano, Turin, Italy; 5 CTO Andrea Alesini, Rome, Italy; 6 Ospedale San Giuliano, Giugliano, Naples, Italy; 7 Department of Medicine and Health Sciences “Vincenzo Tiberio”, University of Molise, Campobasso, Italy; 8 Ente Ecclesiastico Ospedale Generale F. Miulli, Acquaviva Delle Fonti, Bari, Italy; 9 UO Chirurgia Della Spalla, Cliniche Humanitas Gavazzeni E Castelli, Bergamo, Italy; 10 University of Modena and Reggio Emilia, Modena, Italy; 11 Department of Medical and Surgical Specialties, Radiological Sciences, and Public Health, University of Brescia, Brescia, Italy; 12 Department of Bone and Joint Surgery, Spedali Civili, Brescia, Italy; 13 University La Sapienza, Rome, Italy; 14 Department of Orthopaedic and Traumatology Surgery, Shoulder and Elbow Unit, Hesperia Hospital Modena, Modena, Italy

**Keywords:** TikTok, Social media, Reels, Epicondylitis, Elbow, Sports medicine, Rehabilitation

## Abstract

**Purpose:**

This study aimed to assess the validity and informational value of TikTok content about epicondylitis. The hypothesis tested herein was that TikTok video content would not provide adequate and valid information.

**Methods:**

The term “epicondylitis” was used as a keyword to comprehensively search for TikTok videos, and the first 100 videos that were retrieved were subsequently included for analysis. The duration, number of likes, number of shares and number of views were recorded for each video. Furthermore, the videos were categorized on the basis of their source (medical doctor, physiotherapist, or private user), type of information (physical therapy, anatomy, clinical examination, etiopathogenesis, patient experience, treatment, or other), video content (rehabilitation, education, or patient experience/testimony), and the presence of music or voice. Assessments of video content quality and reliability were conducted using the DISCERN tool, the Journal of the American Medical Association (JAMA) benchmark criteria, and the Global Quality Score (GQS).

**Results:**

A total of 100 videos were included in the analysis: 78 (78.0%) were published by physiotherapists, 18 were published by medical doctors (18.0%), and 4 were published by private users (4.0%). Most of the information pertained to physical therapy (75; 75.0%) and most of the content was about rehabilitation (75; 75.0%). The mean length of the videos was 42.51 ± 24.75 seconds; the mean number of views was 193,207.78 ± 1,300,853.86; and the mean number of comments, likes, and shares were 22.43 ± 62.54, 1578.52 ± 8333.11, and 149.87 ± 577.73, respectively. The mean DISCERN score, JAMA score, and GQS were 18.12 ± 5.73, 0.80 ± 0.53, and 1.30 ± 0.52, respectively. Videos posted by medical doctors/private users had higher scores (*p* < 0.05) than videos posted by physiotherapists. Videos that focused on education or patient experience had higher scores (*p* < 0.05) than videos based on rehabilitation.

**Conclusions:**

TikTok can be an unreliable source of information regarding epicondylitis treatment. It is common to find nonphysicians who share medical advice on the platform, with medical treatments demonstrating the weakest level of supporting evidence. Elbow surgeons should advise their patients that treatment recommendations from TikTok may not align with established guidelines.

*Level of Evidence*: Level IV—Cross-sectional study.

## Introduction

Lateral epicondylitis, also referred to as “tennis elbow,” is a musculoskeletal disorder that affects approximately 1–3% of the overall population. It primarily affects individuals above the age of 40, with an equal distribution between the two sexes. The majority of previous studies suggest that 70–90% of lateral epicondylitis patients experience spontaneous remission or respond well to conservative treatment after 1 year [1]. This treatment typically includes rest, nonsteroidal anti-inflammatory medicines, orthosis, physical treatments, and injections. Several recent studies have demonstrated that injections of corticosteroids, platelet-rich plasma, autologous blood products, or botulinum toxin effectively alleviate pain and improve functionality in patients with lateral epicondylitis who are unresponsive to pain medications and are seeking to avoid surgery [2].

Owing to the excessive tension, repetitive microtrauma, and degenerative alterations of the extensor carpi radialis brevis (ECRB) tendon, both arthroscopic and open surgery are commonly recommended for patients with lateral epicondylitis who are unlikely to respond well to conservative treatment [3]. There is ongoing debate regarding the indications for surgical treatment among patients with epicondylitis. However, surgical intervention, particularly arthroscopic techniques, is typically beneficial for patients who continue to experience debilitating pain even after 6 months of nonoperative treatment. Arthroscopic techniques offer advantages, such as enhanced visualization of components within the joint, a shorter recovery period, and fewer complications after surgery [4].

The remarkable expansion of internet-based medical information has profoundly altered the manner in which individuals acquire health-related knowledge. In contemporary times, an increasing number of patients search for information on the internet prior to scheduling an appointment with a medical professional [5]. Owing to a surge in video content, videos have emerged as a crucial medium for individuals to acquire medical knowledge. Nevertheless, the standard of health-related videos available on the internet is considerably unsatisfactory. There is ample opportunity to enhance the quality of health-related videos [6]. While there has been extensive research on health materials available on video platforms, such as YouTube, there is still a lack of research on the same content on emerging short video apps, such as TikTok. TikTok is accessible in more than 150 countries, boasts a user base exceeding 1 billion, and has been downloaded more than 200 million times in the US alone. TikTok allows users to generate their own videos by lip-synching or engaging in dance routines to popular music tracks. TikTok encompasses not only entertainment but also a plethora of health care-related content. According to a recent study, TikTok has the potential to serve as a significant platform for consumers to obtain and embrace health-related information. Furthermore, TikTok possesses significant potential to enhance public health communication. Scientists have investigated the visual clarity of TikTok videos of orthopedic procedures related to the anterior cruciate ligament, scoliosis, and osteoarthritis [7–10].

This study aimed to assess the validity and informational value of TikTok content for the treatment of epicondylitis. The hypothesis tested herein was that the platform video content would not provide adequate and valid information.

## Methods

The current study was exempt from institutional review board approval. This study focused on epicondylitis-related videos on TikTok. The term “epicondylitis” was used as a keyword for an extensive TikTok video search on 5 November 2023, and the first 100 videos were recorded. Out-of-topic, non-English, and duplicated videos were excluded from the analysis.

The duration and number of likes, shares, and views of each video were recorded. Furthermore, the videos were categorized on the basis of their source (medical doctor, physiotherapist, or private user), type of information (physical therapy, anatomy, clinical examination, etiopathogenesis, patient experience, treatment, or other), video content (rehabilitation, education, or patient experience/testimony), and the presence of music or voice.

The video content quality and reliability were assessed by two experienced shoulder-and-elbow surgeons using the DISCERN instrument, the Journal of the American Medical Association (JAMA) benchmark criteria, and the Global Quality Score (GQS) [11–18].

### Assessment tools for video reliability, validity, and quality

- DISCERN instrument

The DISCERN tool is an assessment scale developed for patients and providers to assess the reliability and quality of information. The tool, which consists of 16 items in total, is divided into three parts. Items 1 through 8 form the first part and measure the reliability of the information. Items 9 through 15 form the second part, measuring the quality of the information, and the last section (item 16) consists of a single item providing an overall quality rating. DISCERN uses a five-point Likert scale. For the evaluation of the first 15 items, a score of 1 indicates “no” and a score of 5 indicates “yes”. For item 16, a score of 1 indicates “low quality with serious or extensive deficiencies” and a score of 5 indicates “high quality with minimum-wax deficiencies.” The total DISCERN score was calculated as the sum of the first 15 items, ranging from a minimum score of 15 to a maximum score of 75. The higher the score, the greater the reliability and quality of the information—a score of 15–27 points indicate “very poor,” 28–38 points indicate “poor,” 39–50 points indicate “medium,” 51–62 points indicate “good,” and 63–75 points indicate “excellent.” The DISCERN tool is freely accessible at http://www.discern.org.uk [11, 12, 16].

- JAMA benchmark criteria

The JAMA benchmark criteria instrument is one of the leading tools used to evaluate medical information obtained from online sources. It includes four criteria, namely, authorship, attribution, disclosure, and currency, with a maximum value of one point each and a maximum possible total score of 4 points. In the JAMA evaluation, a score of 0–1 points represents insufficient information, a score of 2–3 points represents partially sufficient information, and a score of 4 points represents completely sufficient information [17, 18].

- Global quality score

The GQS is a scoring system for assessing a video in terms of its instructive aspects. It allows for the evaluation of the quality, streaming, and usability of information presented in online videos. In the evaluation of the GQS, a score of 1 indicates that the video has the poorest quality and is not useful for viewers, while a score of 5 indicates that the video has excellent quality and is very useful for viewers [13, 14].

### Statistical analysis

Descriptive statistics were presented for all video characteristics, including video sources, video content, audio, type of video information, and outcomes (i.e., DISCERN, JAMA, and GQS). Categorical variables are shown as absolute frequencies and percentages, while continuous variables are presented as the mean and standard deviation or median, interquartile range (IQR), and range.

Correlations between quantitative variables were estimated and tested using the Spearman rank correlation test, while the normality of continuous variables was assessed with the Shapiro‒Wilk test. A Wilcoxon Mann‒Whitney test was performed to evaluate whether outcomes differed by video sources, audio, information type, and video content. For the analyses described above, some categories of video sources (doctor and private user), information types (anatomy, clinical examination, etiopathogenesis, patient experience, treatment, and other), and video content (education or patient experience/testimony) were grouped into one category owing to their low frequency. All tests were two-tailed, and a *p* value < 0.05 was considered to indicate statistical significance. All the statistical tests were performed with R (R Foundation for Statistical Computing, Vienna, Austria; URL: https://www.R-project.org/).

## Results

A total of 100 videos were included in the analysis: 78 (78.0%) were published by physiotherapists, 18 by medical doctors (18.0%), and 4 by private users (4.0%). Most of the information pertained to physical therapy (75; 75.0%), followed by anatomy (11; 11.0%), clinical examination (5; 5.0%), etiopathogenesis (5; 5.0%), patient experience (2; 2.0%), treatment (1; 1.0%), and other (1; 1.0%).

The video content included rehabilitation in 75 (75.0%) videos, education in 22 (22.0%) videos, and patient experience/testimony in 3 (3.0%) videos. In total, 36 (36.0%) videos used music as the audio background, and 64 (64.0%) had voice comments. The detailed results are reported in Table 1.

**Table 1:** Categorical variables

	*N* = 100
	*n* (%)
Video source	
Doctor	18 (18.0)
Physiotherapist	78 (78.0)
Private user	4 (4.0)
Company	0 (0.0)
Type of information	
Physical therapy	75 (75.0)
Anatomy	11 (11.0)
Clinical examination	5 (5.0)
Etiopathogenesis	5 (5.0)
Patient experience	2 (2.0)
Treatment	1 (1.0)
Other	1 (1.0)
Video content	
Rehabilitation	75 (75.0)
Education	22 (22.0)
Patient experience/testimony	3 (3.0)
Audio characteristics	
Music	36 (36.0)
Voice	64 (64.0)

The mean length of the videos was 42.51 ± 24.75 s; the mean number of views was 193,207.78 ± 1,300,853.86; and the mean numbers of comments, likes, and shares were 22.43 ± 62.54, 1578.52 ± 8333.11, and 149.87 ± 577.73, respectively. The mean DISCERN score, JAMA score, and GQS were 18.12 ± 5.73, 0.80 ± 0.53, and 1.30 ± 0.52, respectively. The detailed results are reported in Table 2.

**Table 2:** Continuous variables

	*N* = 100	
	Mean ± SD	Median [IQR; range]
Video characteristics		
Total number of views	193,207.78 ± 1,300,853.86	10,650 [1993–48,550; 101–12,800,000]
Total number of likes	1578.52 ± 8333.11	215.50 [55.75–1030.50; 3–83,100]
Total number of comments	22.43 ± 62.54	5 [1–21.25; 0–459]
Total number of shares	149.87 ± 577.73	16.50 [3–73.50; 0–5492]
Video length (s)	42.51 ± 24.75	39 [22–58.25; 5–129]
Video score		
DISCERN	18.12 ± 5.73	16 [15–19; 15–45]
JAMA	0.80 ± 0.53	1 [0–1; 0–2]
GQS	1.30 ± 0.52	1 [1–2; 1–3]

*IQR*  interquartile range; *SD* standard deviation

### Significant correlations

The only significant correlations (*p* < 0.05) were found between video length and DISCERN and JAMA scores. The results are reported in Table 3.

**Table 3:** Correlations between scores and video characteristics

	DISCERN		JAMA		GQS	
	ρ	*p* value	ρ	*p* value	ρ	*p* value
Total number of views	−0.09	0.379	0.00	0.978	0.04	0.678
Total number of likes	−0.11	0.295	−0.04	0.698	−0.02	0.853
Total number of shares	0.04	0.684	0.12	0.217	0.06	0.582
Total number of comments	−0.04	0.716	−0.07	0.472	−0.01	0.942
Video length (s)	0.26	0.009*	0.28	0.005*	0.11	0.294

*N* = 100, *ρ* estimated using Spearman rank correlation

^*^Statistically significant value

The number of views was positively correlated with the number of likes, shares, and comments (*p* < 0.05), the number of shares was positively correlated with the number of likes and comments (*p* < 0.05), and the number of likes was positively correlated with the number of comments (*p* < 0.05). The detailed results are reported in Table 4.

**Table 4:** Correlation between the characteristics of the videos

Variables		*ρ*	*p* value
Total number of views	Total number of likes	0.94	< 0.001*
Total number of shares	Total number of views	0.87	< 0.001*
Total number of shares	Total number of likes	0.84	< 0.001*
Total number of likes	Total number of comments	0.81	< 0.001*
Total number of views	Total number of comments	0.74	< 0.001*
Total number of shares	Total number of comments	0.68	< 0.001*
Total number of comments	Video length (s)	0.02	0.845
Total number of shares	Video length (s)	0.00	0.998
Total number of likes	Video length (s)	0.00	0.966
Total number of views	Video length (s)	−0.05	0.599

*N* = 100, *ρ* estimated using Spearman rank correlation

^*^Statistically significant value

### Video comparisons

#### Video source

All the scores for videos posted by medical doctors/private users were higher (*p* < 0.05) than those for videos posted by physiotherapists. The detailed results are reported in Table 5.

**Table 5:** Score difference by video sources

	Physiotherapist	Doctor or private user	*p* value
	*N* = 78	*N* = 22	
	Mean ± SD	Mean ± SD	
DISCERN	16.28 ± 1.92	24.64 ± 9.19	< 0.001*
JAMA	0.72 ± 0.45	1.09 ± 0.68	0.010*
GQS	1.15 ± 0.36	1.82 ± 0.66	< 0.001*

^a^“Doctor” and “Private user” were grouped in one category owing to their low frequency

^*^Statistically significant value

### Type of information

Other videos (including anatomy, clinical examination, etiopathogenesis, patient experience, treatment, and others) had higher scores (*p* < 0.05) than videos based on physical therapy. The detailed results are reported in Table 6.

**Table 6:** Score difference by type of information

	Physical therapy	Other^a^	*p* value
	*N* = 75	*N* = 25	
	Mean ± SD	Mean ± SD	
DISCERN	16.17 ± 1.83	23.96 ± 8.83	< 0.001*
JAMA	0.71 ± 0.46	1.08 ± 0.64	0.006*
GQS	1.13 ± 0.34	1.80 ± 0.65	< 0.001*

^a^“Anatomy,” “clinical examination,” “ethiopatogenesis,” “patient experience,” “treatment,” and “other” were grouped in one category owing to their low frequency

^*^Statistically significant value

### Video content

Videos focused on education or patient experience reported higher values for all the scores (*p* < 0.05) than videos based on rehabilitation. The detailed results are reported in Table 7.

**Table 7:** Score difference by video content

	Rehabilitation	Education or patient experience/testimony	*p* value
	*N* = 75	*N* = 25	
	Mean ± SD	Mean ± SD	
DISCERN	16.17 ± 1.83	23.96 ± 8.83	< 0.001*
JAMA	0.71 ± 0.46	1.08 ± 0.64	0.006*
GQS	1.13 ± 0.34	1.80 ± 0.65	< 0.001*

^a^“Education” and “patient experience/testimony” were grouped in one category owing to their low frequency

^*^Statistically significant value

### Audio

Videos with a voice showed higher scores than videos with a music background (*p* < 0.05). The detailed results are reported in Table 8.

**Table 8:** Analysis by audio background

	Music	Voice	*p* value
	*N* = 36	*N* = 64	
	Mean ± SD	Mean ± SD	
DISCERN	15.36 ± 0.99	19.67 ± 6.66	< 0.001*
JAMA	0.44 ± 0.50	1.00 ± 0.44	< 0.001*
GQS	1.06 ± 0.23	1.44 ± 0.59	< 0.001*

^*^Statistically significant value

## Discussion

The main findings of this cross-sectional study demonstrate that epicondylitis-themed TikTok videos were mainly published by physiotherapists and the main focus was rehabilitation and physical therapy. Most videos had low educational value, and videos posted by medical doctors showed high educational value for all scores (JAMA score, GQS, and DISCERN score).

Currently, the amount of scientific literature on new social media platforms, such as TikTok is growing. However, there is a paucity of research on orthopedic diseases and pathologies. All available articles presented results similar to ours, with very low scores and educational value.

Tabarestani et al. recently assessed the quality and educational benefits of Achilles tendinopathy-related TikTok videos and found that although TikTok is a powerful tool for information distribution, the educational value of TikTok videos related to Achilles tendinopathy exercises was poor, with only 1% of videos receiving a grade of “fair” and no videos receiving a score of “good” or “excellent.” Similarly, Hong et al. recently identified the creators of knee osteoarthritis-related content on TikTok and examined whether a connection exists between the reach of video content and the strength of the recommendations provided. The authors concluded that TikTok can be unreliable for knee osteoarthritis treatment and that much of the information was posted by nonphysicians who shared medical advice [19].

Kolade et al. aimed to evaluate the accuracy and popularity of content on common orthopedic pathology on TikTok and Instagram. There were 165,666,490 views on TikTok and 9,631,015 views on Instagram among the six common orthopedic conditions (Achilles tendon tear, anterior cruciate ligament [ACL] tear, meniscus tear, tennis elbow, rotator cuff tear, and ankle sprains). The content created by physicians had less overall engagement (16.1%) than the content created by nonphysicians (83.9%). On average, the quality of the content was low. Physician-created posts were significantly more accurate than nonphysician-created posts. Common orthopedic conditions, such as Achilles tendon tears, ACL tears, and meniscus tears, are frequently the focus of TikTok and Instagram videos; however, this information is often not medically accurate [20].

One aspect that should be considered is that almost all of the videos are posted by physical therapists and are about rehabilitative exercises and physical therapies; however, the optimal management of lateral epicondylitis in high-functioning patients remains unclear. Despite a lack of high-level evidence to inform clinical decision-making, nonoperative management represents a first-line treatment. Although there is consensus that nonoperative management should represent first-line treatment, guidelines informing the optimal approach to nonsurgical treatment are not well established [21]. Evidence is lacking regarding the superiority of one nonoperative treatment option over another, and past systematic reviews have not reached definitive conclusions [22, 23]. It is difficult to determine how a platform such as TikTok can guarantee scientific evidence not found in literature, especially when the quality of rehabilitation videos reports little or no educational value.

Bethell et al. analyzed the quality, reliability, and educational value of TikTok videos among the ACL injury patient population. A total of 111 videos with 5,520,660 cumulative views were examined. Of these videos, 84 and 27 were created by the general public and health care professionals, respectively. The differences in the DISCERN and the ACL exercise education (ACLEES) scores between general users and health care professionals were not statistically significant. Compared with the general public, health care professionals had a greater percentage of videos with a ‘‘very poor’’ DISCERN score (66.67% versus 53.57%, respectively). The overall educational value of the TikTok videos related to ACL rehabilitation exercises was very poor [8].

In 2023, Anastastio et al. assessed the quality and educational benefits of ankle sprain-related TikTok videos and confirmed that the educational value of the videos related to ankle sprain injury exercises was poor. With only 2% of videos receiving a grade of “fair” and no videos reaching a score of “good” or “excellent,” health care professionals should be aware of the low-quality content easily accessible on TikTok [24].

Similarly, Jang et al. explored videos introducing scoliosis exercises on TikTok, highlighting how the overall information quality, reliability, and educational suitability of these videos appear to be low, suggesting that TikTok is not a suitable source for obtaining scoliosis exercise information. A similar article published by Bethell reported that the overall educational value of videos related to shoulder instability exercises was poor [25].

Social media platforms have undergone significant expansion and varying degrees of user involvement over time. The primary platforms used by customers in 2023 are Facebook (69%), YouTube (57%), Instagram (45%), TikTok (33%), and Twitter (30%). The worldwide social media user population has shown a consistent upward trend, increasing from 4.2 billion users in January 2021 to 4.62 billion users by January 2022, indicating a year-over-year growth of 10.1%. As of January 2023, there has been a moderate increase of 3%, resulting in the addition of 137 million users [26].

Social media platforms have become essential instruments in the health care industry, serving vital functions in medical research, education, patient communication, professional growth, and the dissemination of health-related information. Social media platforms have been prominent in several health fields, including vaccinations, medications, smoking, noncommunicable illnesses, pandemics, eating disorders, and medical treatments, as a substantial number of people use these platforms to search for and exchange health information. Social media has provided various health-related applications that have greatly aided medical research. These applications encompass health interventions, health campaigns, medical education, and disease outbreak surveillance. Moreover, considerable attention has been given to the correlation between the utilization of social media and mental well-being, suggesting its potential as a platform for aiding individuals with mental health problems [27].

Social media has become a popular source of medical education, with health care professionals and organizations increasingly utilizing this tool. The use of media devices by younger people suggests that social media can be crucial in delivering medical education to future health care workers. Social media empowers users to generate and distribute content, interact with others in social networks, and engage in active learning, hence promoting introspection and knowledge creation. Additional empirical research is necessary to ascertain the instructional usefulness of social media in medical education, notwithstanding its potential benefits. Medical education curricula currently lack thorough guidance on the purposeful utilization and implementation of social media. Health care professionals and organizations need to adapt and accept social media platforms as a means for medical education while maintaining the same ethical guidelines as they would in face-to-face patient encounters [26].

There are limitations to this study. Geographic location and user attributes can potentially impact the outcomes of the search algorithm. The analysis did not include videos that were not in English, thereby decreasing the generalizability of the findings. Furthermore, the present study employed reliability, validity, and quality evaluation instruments, namely the DISCERN score, JAMA score, and the GQS, that have not been completely validated. Nevertheless, these tools are extensively employed in research endeavors aimed at assessing the efficacy of these metrics for online services.

## Conclusions

TikTok can be an unreliable source of epicondylitis treatment information. It is common to find nonphysicians who share medical advice on the platform, with medical treatments demonstrating the weakest level of supporting evidence. Elbow surgeons should advise their patients that treatment recommendations from TikTok may not align with established guidelines.

## Data Availability

Raw data are available upon request to the corresponding author.

## References

[1] Ahmed AF, Rayyan R, Zikria BA, Salameh M (2023). Lateral epicondylitis of the elbow: an up-to-date review of management. Eur J Orthop Surg Traumatol.

[2] Vaquero-Picado A, Barco R, Antuña SA (2017). Lateral epicondylitis of the elbow. EFORT Open Rev.

[3] Kim GM, Yoo SJ, Choi S, Park YG (2019). Current trends for treating lateral epicondylitis. Clin Shoulder Elb.

[4] Li Y, Liu F, Badre A (2022). Lateral epicondylosis. Can Med Assoc J.

[5] Elhajjar S, Ouaida F (2022). Use of social media in healthcare. Health Mark Q.

[6] Moorhead SA, Hazlett DE, Harrison L, Carroll JK, Irwin A, Hoving C (2013). A new dimension of health care: systematic review of the uses, benefits, and limitations of social media for health communication. J Med Internet Res.

[7] Yeung AWK, Tosevska A, Klager E, Eibensteiner F, Tsagkaris C, Parvanov ED, Nawaz FA, Völkl-Kernstock S, Schaden E, Kletecka-Pulker M, Willschke H, Atanasov AG (2022). Medical and health-related misinformation on social media: bibliometric study of the scientific literature. J Med Internet Res.

[8] Bethell MA, Anastasio AT, Adu-Kwarteng K, Tabarestani TQ, Lau BC (2023). Analyzing the quality, reliability, and educational value of ACL rehabilitation exercises on TikTok: a cross-sectional study. Orthop J Sports Med.

[9] Aflatooni JO, Loving R, Holderread BM, Liberman SR, Harris JD (2023). #Scoliosis: an analysis of patient perception of scoliosis on TikTok. Proc (Bayl Univ Med Cent).

[10] Hong TI, Bernstein SL, Ramirez A, Gu A, Agarwal AR, Lutton DM, Tabaie S (2023). Analysis of the perception and treatment of osteoarthritis of the knee through social media: an observational study of the top 100 viral TikTok videos. Cureus.

[11] Charnock D, Shepperd S, Needham G, Gann R (1999). DISCERN: an instrument for judging the quality of written consumer health information on treatment choices. J Epidemiol Community Health.

[12] DISCERN Website. http://www.discern.org.uk/discern_instrument.php. Accessed 3 Nov 2023

[13] Erdem MN, Karaca S (2018). Evaluating the accuracy and quality of the information in Kyphosis videos shared on YouTube. Spine.

[14] Silberg WM, Lundberg GD, Musacchio RA (1997). Assessing, controlling, and assuring the quality of medical information on the internet: caveant lector et viewor–Let the reader and viewer beware. JAMA.

[15] Śledzińska P, Bebyn MG, Furtak J (2021). Quality of YouTube videos on meningioma treatment using the DISCERN instrument. World Neurosurg.

[16] Szmuda T, Alkhater A, Albrahim M, Alquraya E, Ali S, Dunquwah RA, Słoniewski P (2020). YouTube as a source of patient information for stroke: a content-quality and an audience engagement analysis. J Stroke Cerebrovasc Dis.

[17] Yurdaisik I (2020). Analysis of the most viewed first 50 videos on YouTube about breast cancer. Biomed Res Int.

[18] Zhang S, Fukunaga T, Oka S, Orita H, Kaji S, Yube Y, Yamauchi S, Kohira Y, Egawa H (2020). Concerns of quality, utility, and reliability of laparoscopic gastrectomy for gastric cancer in public video sharing platform. Ann Transl Med.

[19] Tabarestani TQ, Anastasio AT, Duruewuru A, Taylor JR, Bethell MA, Adams SB (2023). Analyzing the quality and educational value of Achilles tendinopathy-related videos on TikTok. Foot Ankle Surg.

[20] Kolade O, Martinez R, Awe A, Dubin JM, Mehran N, Mulcahey MK, Tabaie S (2023). Misinformation about orthopaedic conditions on social media: analysis of TikTok and Instagram. Cureus.

[21] Chen Q, Shen P, Zhang B, Chen Y, Zheng C (2023). Long-term effectiveness of conservative management for lateral epicondylitis: a meta-analysis. J Plast Surg Hand Surg.

[22] Sims SE, Miller K, Elfar JC, Hammert WC (2014). Non-surgical treatment of lateral epicondylitis: a systematic review of randomized controlled trials. Hand.

[23] Jeon JY, Lee MH, Jeon IH, Chung HW, Lee SH, Shin MJ (2018). Lateral epicondylitis: associations of MR imaging and clinical assessments with treatment options in patients receiving conservative and arthroscopic managements. Eur Radiol.

[24] Anastasio AT, Tabarestani TQ, Bagheri K, Bethell MA, Prado I, Taylor JR, Adams SB (2023). A new trend in social media and medicine: the poor quality of videos related to ankle sprain exercises on TikTok. Foot Ankle Orthop.

[25] Jang CW, Kim M, Kang SW, Cho HE (2022). Reliability, quality, and educational suitability of TikTok videos as a source of information about scoliosis exercises: a cross-sectional study. Healthcare.

[26] Kanchan S, Gaidhane A (2023). Social media role and its impact on public health: a narrative review. Cureus.

[27] Joseph J, Varghese A, Vr V, Dhandapani M, Grover S, Sharma S, Khakha D, Mann S, Varkey BP (2021). Prevalence of internet addiction among college students in the Indian setting: a systematic review and meta-analysis. Gen Psychiatr.

